# Systematic elucidation of the mechanism of Jingyin granule in the treatment of Novel Coronavirus (COVID-19) Pneumonia via Network Pharmacology

**DOI:** 10.7150/ijms.53575

**Published:** 2021-02-05

**Authors:** Bingrui Wang, Xuehua Sun, Xiaoni Kong, Yueqiu Gao

**Affiliations:** 1 Institute of Infection Disease, Department of Liver Diseases, Central Laboratory, ShuGuang Hospital affiliated to Shanghai University of Chinese Traditional Medicine, Shanghai, China.; 2 Department of Liver Surgery, Renji Hospital, School of Medicine, Shanghai Jiao Tong University, Shanghai, China.

**Keywords:** Jingyin granule, ingredient, druggability, network pharmacology

## Abstract

**Background:** Jingyin granule is one of the widely used traditional Chinese medicine mixture composed of multiple herbs in the treatment of respiratory system diseases. The mechanism of its therapeutic effects has still been obscure. The aim of this study is to use the network pharmacology approach for identification of the main active ingredients of Jingyin granule against COVID-19 targets and to explore their therapeutic mechanism.

**Material and Method:** In this study, the ingredients of Jingyin granule were evaluated by the usage of Traditional Chinese Medicine Systems Pharmacology Database and Traditional Chinese Medicine Integrated Database, and the interactions between potential gene targets and ingredients were identified using the SwissTargetPrediction database. Meanwhile the possible efficient targets COVID-19 acts on were identified via Online Mendelian Inheritance in Man database, DisGeNET database and GeneCards database. In addition, functions, components and pathways were identified by Gene Ontology enrichment analysis and Kyoto Encyclopedia of Genes and Genomes pathway analysis. Protein interaction, ingredients-targets network was established.

**Results:** Our findings showed that numerous ingredients of Jingyin granule could act on COVID-19 with 88 target genes. GO enrichment analysis, KEGG pathway analysis, and protein-protein interaction network revealed that these targets were interrelated with regulation of immune function, directly targeting disease genes.

**Conclusions:** Jingyin granule could be utilized to exert systematic pharmacological effects. Jingyin granule could directly target the major genes, and also regulate the immune system, acting as oblique disease treatment.

## Introduction

Early in December 2019, an atypical pneumonia became epidemic in Wuhan, China 1, 2. On Feb 11, 2020, the world health organization (WHO) announced a new name for the epidemic disease as 2019-new coronavirus disease (COVID-19). The pathogen of COVID-19 has been identified and this novel coronavirus is denominated as severe acute respiratory syndrome coronavirus 2 (SARS-CoV-2). Coronavirus are a category of special viruses that could cause not only common cold, but also some more severe diseases, include severe acute respiratory syndrome (SARS) and Middle East respiratory syndrome (MERS). There were 79.5% similar genome sequences of SARS-CoV-2 compared to severe acute respiratory syndrome-related coronaviruses (SARS-CoV) 3, 4. Furthermore, a common vital protein of SARS-CoV-2 and SARS-CoV, the spike protein, binds to angiotensin-converting enzyme 2 (ACE2) receptor through the N-terminal S1 subunit which is subsequently cleaved by the host transmembrane serine protease 2 (TMPRSS2) to expose the C-terminal S2 subunit, and then human alveolar epithelial cells are infected 3, 5. COVID-19 is mainly manifested as fever, cough, respiratory difficulty or even respiratory failure in severe cases 6. It is highly infectious and spreads rapidly throughout China and other countries 7, 8, and in traditional Chinese medicine these kinds of diseases were named Wenyi (pestilence).

In the treatment of COVID-19, the combination of Chinese and western medicine has achieved good clinical efficacy in China 9, 10. Jingyin granule, the in-hospital preparation of Shanghai Shuguang hospital, was written into the “Shanghai COVID-19 Chinese medicine treatment program (trial second edition)”, which offers the guidance of the lung syndrome of mild wind-heat (fever or no fever, or aversion to cold, sore throat, cough, little sputum, red tongue, thin or yellow fur, floating and rapid pulse). Jingyin granule had also achieved good clinical effect on COVID-19 in Raytheon hospital in Wuhan.

Jingyin granule has been used in Shuguang hospital affiliated to Shanghai University of traditional Chinese medicine for more than 40 years for lung-wind-heat cold, acute bronchitis and acute pneumonia. It is safe and effective for the respiratory diseases of wind-heat syndrome with fever, aversion to cold, sore throat and cough as the main symptoms. It has also won numerous battles against the epidemic such as SARS, human influenza A (H1N1) infection. Jingyin granule can be utilized as a mixture of valuable chemical probes for the investigation of complex biological processes and identification of potential therapeutic target genes or molecules. However, the particular molecular mechanisms exploring how Jingyin granule takes effects are rarely studied.

Network computational methods are now utilized not only in structure-based drug design and pharmaceutical development, but also in better comprehending the mechanism of how an ingredient of compounds act at the molecular biology level and in this way to discover the therapeutic effect. Nevertheless, because of the prodigious amount, it is impractical to experimentally screen all possible interactions between ingredients of compounds and target proteins. Therefore, computational modelling and network approaches are considered to be notably theoretical methods which can also accurately identify potential drug-target interactions in order to provide evidence for basic experimental studies 11, 12.

In this study, the pharmacological effects and machanisms of Jingyin granule were elucidated systematically by using computational methods. First, the ingredients of Jingyin granule was evaluated by the usage of the Traditional Chinese Medicine Systems Pharmacology Database (TCMSP) 13 and Traditional Chinese Medicine Integrated Database (TCMID) 14. Next the potential therapeutic targets of these ingredients were identified using the Swiss Target Prediction 15, while the possible targets of COVID-19 were identified using Online Mendelian Inheritance in Man (OMIM) database 16, DisGeNET database 17 and GeneCards database 18. In addition, with the list of identified targets, Gene Ontology enrichment analysis and KEGG pathway analysis were investigated. Ultimately, complete networks, involved by ingredient-target network and protein-protein network, were established to offer a summary of the effects and mechanisms of how Jingyin granule acts in COVID-19.

## Materials and methods

### Ingredients collection and druggability assessment

TCMSP database and TCMID database are resources of system pharmacology for the Chinese medicines or relavant compounds which are both informative about identified ingredients of herbs. TCMSP database also provide detail information of the ingredients, include characters of absorption, distribution, metabolism, and excretion (ADME).

Among all the ADME properties, oral bioavailability (OB) and drug likeness (DL) are the foremost features of orally medicines because of their vital roles in assessing the velocity and percentage of the oral drug absorbed and reaching the systemic circulation. In this study, each herb of Jingyin granule was searched to find out identified ingredients in both databases, and all identified ingredients were searched in TCMSP database to screen potential druggable ingredients following the criteria: 1) OB ≥ 30%; 2) DL ≥ 0.18 19, 20.

### Ingredients and disease target genes acquisition

Because of the similarity and homology of SARS-COV-2, MERS-COV and SARS-COV, target genes of diseases caused by these three viruses were searched and screened by GeneCard database, OMIM database and DisGeNET database. All the search results are merged, and reduplicate targets are removed to acquire all the potential target of the diseases. Target genes of ingredients were searched and screened by SwissTargetPrediction database. Jingyin granule matched the target prediction results of effective ingredients with the searched results of diseases related target genes and intersection targets were collected as the relevant potential targets of Jingyin granule for the treatment of COVID-19, which might be the potential therapeutic target set.

### Gene function and pathway enrichment analysis

GO analysis is widely used for genes and gene products annotating, including molecular functions (MF), biological processes (BP), and cellular components (CC). The Kyoto Encyclopedia of Genes and Genomes (KEGG, http://www.genome.ad.jp/kegg/) database is a common database which provides annotation and visualization of pathways to identify relevant genes with similar functions.

In present study, the R package “clusterProfiler” 21 was utilized to perform GO analysis, and KEGG pathway analysis of the potential therapeutic target set. The top 20 of all terms in every category were listed, and p < 0.05 was set as a significant difference.

### Target gene set analysis by GeneMANIA

GeneMANIA is a web server, which is considered user-friendly and flexible and widely used for finding out the similarity or association of a list of genes 22.

GeneMANIA can aggregate the related genes, which have interactions, similar domains, and analogous functions by inputting a prescribed list. It also shows a network of functional relationships that illustrate the association of the query genes through publicly available data. The potential therapeutic target genes were searched, and the results were visualized by Cytoscape.

## Results

### Pharmacokinetics properties of Jingyin granule

**Figure 1:**
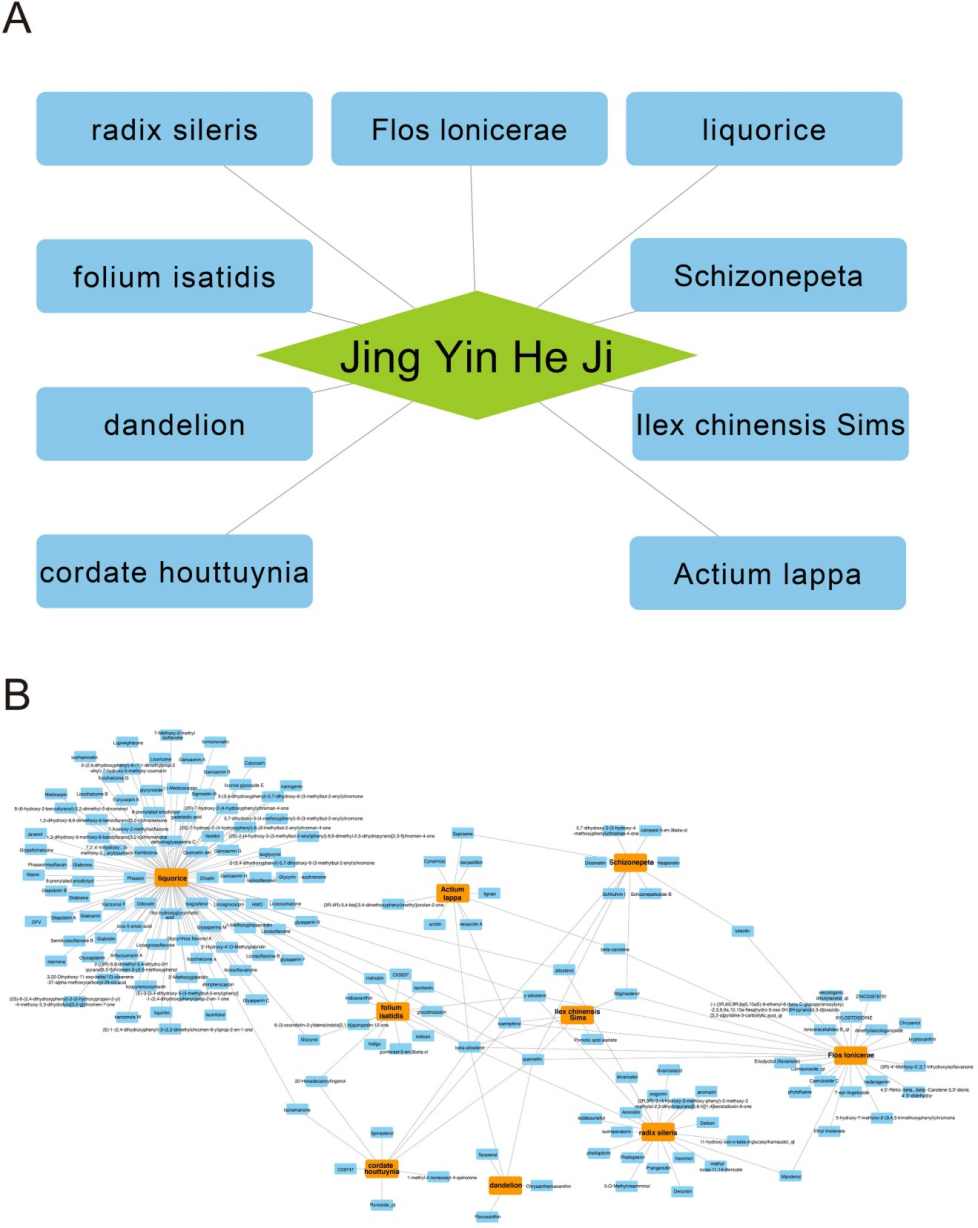
Herb compounds and ingredients of Jingyin granule.

There are 9 herbs consisting of Jingyin granule (Fig. 1A). Absorption, distribution, metabolism and excretion (ADME) are used to describe the configuration of a pharmaceutical compound. TCMSP database can provides information of ADME-related important properties like ACD-calculated partition coefficient (AlogP), hydrogen bond donor/hydrogen bond acceptor (Hdon/Hacc), oral bioavailability (OB), Caco-2 simulated intestinal epithelial permeability (Caco-2), blood brain barrier (BBB), drug likeness (DL) and half-life (HL). By using TCMSP database, AMDE-related properties of Jingyin granule were thoroughly investigated. Ingredients met the following criterion were selected as potential therapeutic drugs: 1) OB ≥ 30%; 2) DL ≥ 0.18. A total of 168 ingredients were selected (Fig. 1B).

### Targets authentication of Jingyin granule and COVID-19

Potential therapeutic targets of the ingredients of Jingyin granule were computational calculated using the SwissTargetPrediction database as described. In total, 865 potential target genes were authenticated by SwissTargetPrediction database.

**Figure 2:**
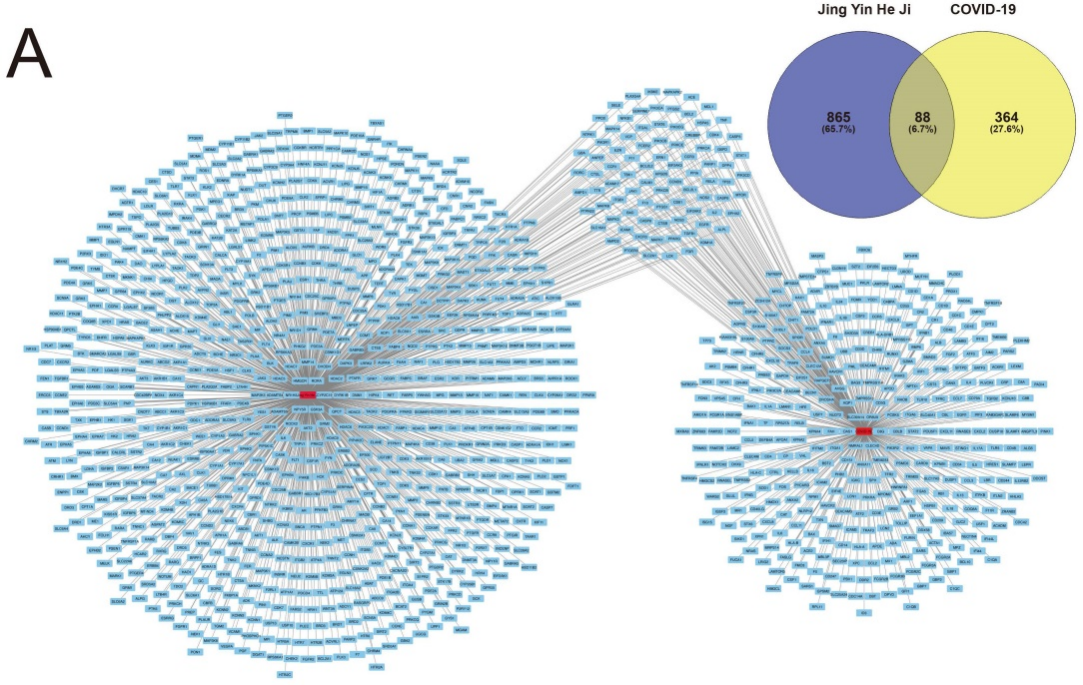
Herb compounds and ingredients of Jingyin granule.

Contemporary, Target genes of COVID-19 were predicted using the GeneCard database, OMIM database and DisGeNET database as described. 364 genes altogether were screened as target genes of COVID-19. The intersection of both selected genes was authenticated as the potential therapeutic target of Jingyin granule to COVID-19, and these 88 identified interacting genes were screened for subsequent investigation (Fig. 2).

### GO enrichment and KEGG pathway analysis

**Figure 3:**
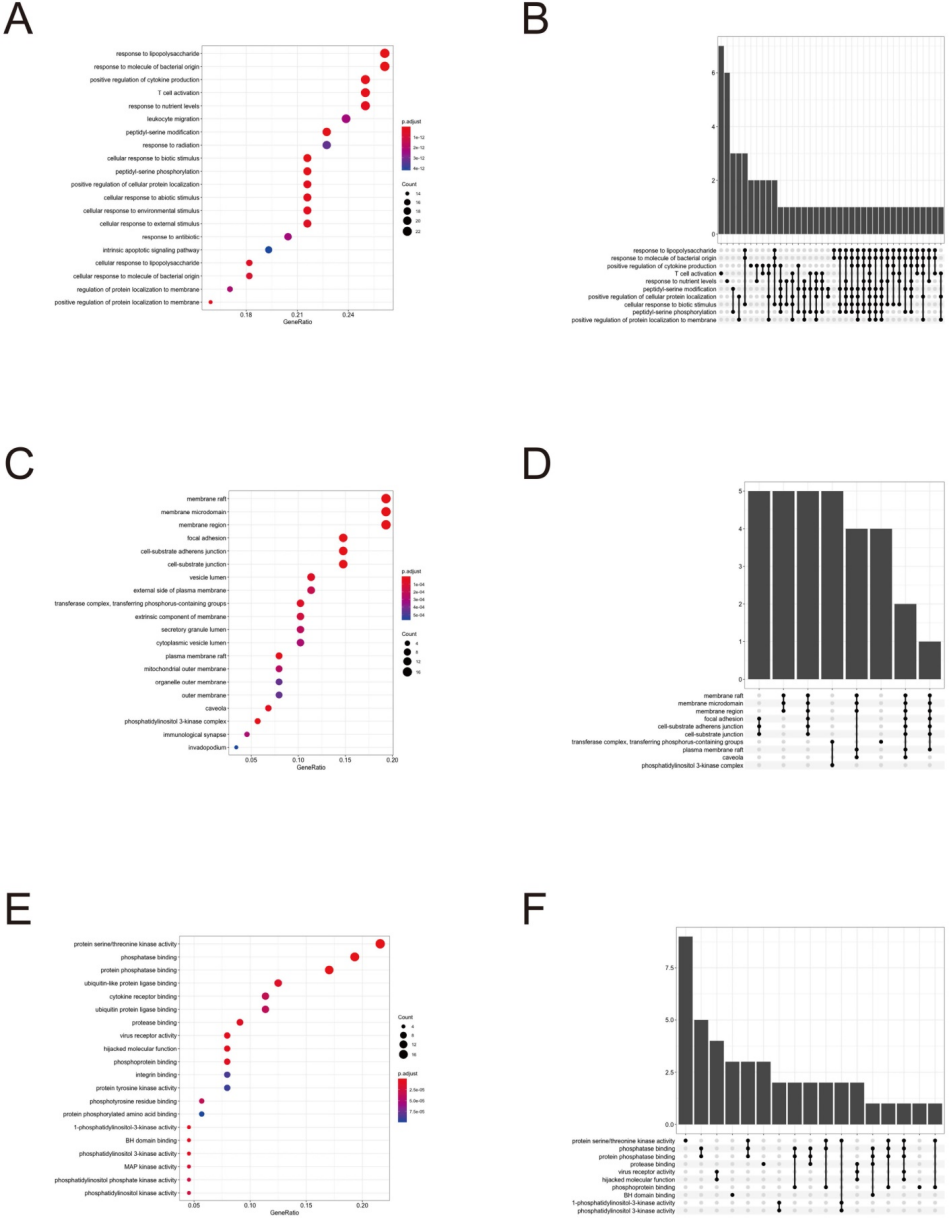
GO enrichment analysis terms of potential therapeutic target genes.

**Figure 4:**
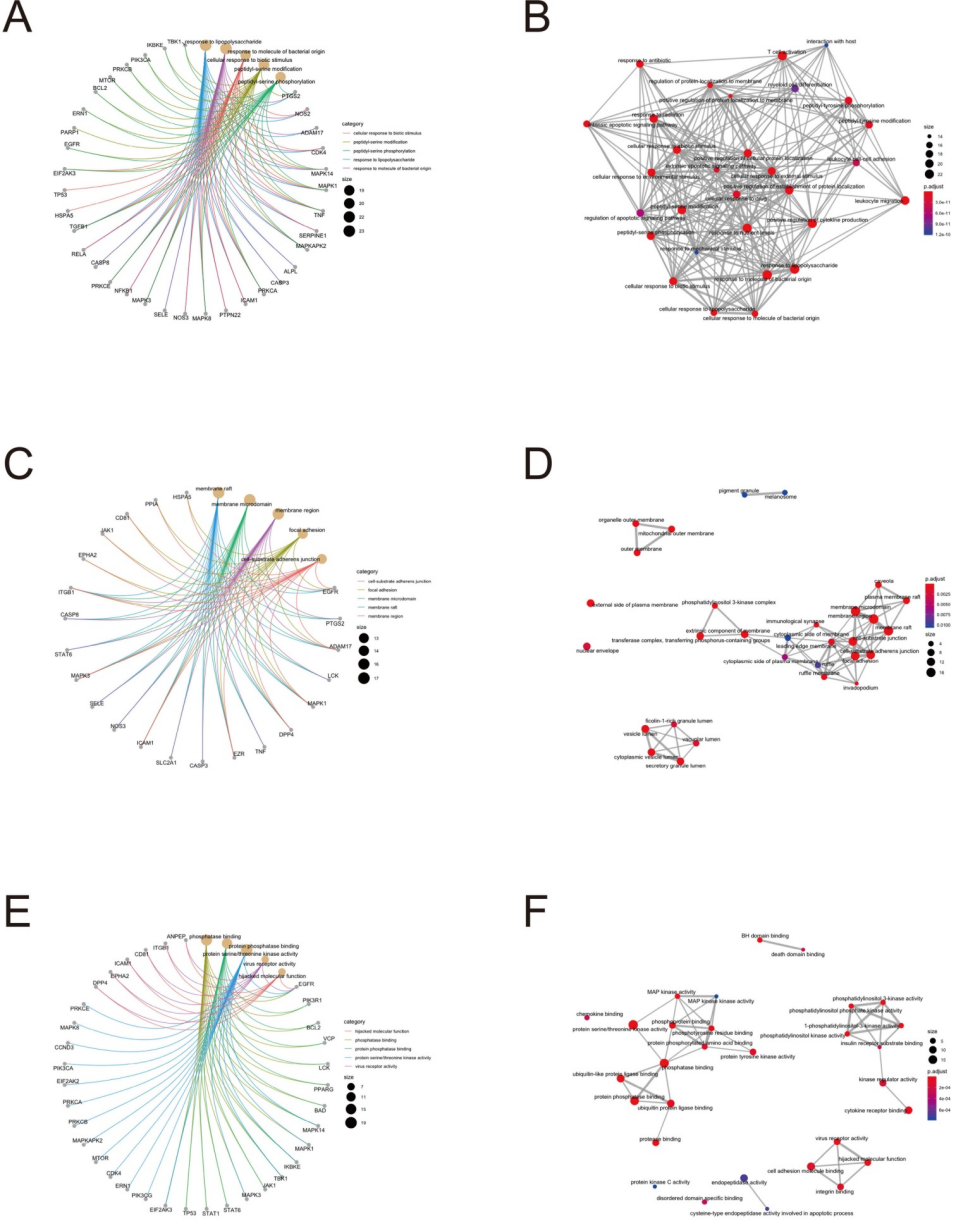
Target gene and term networks of GO enrichment analysis.

**Figure 5:**
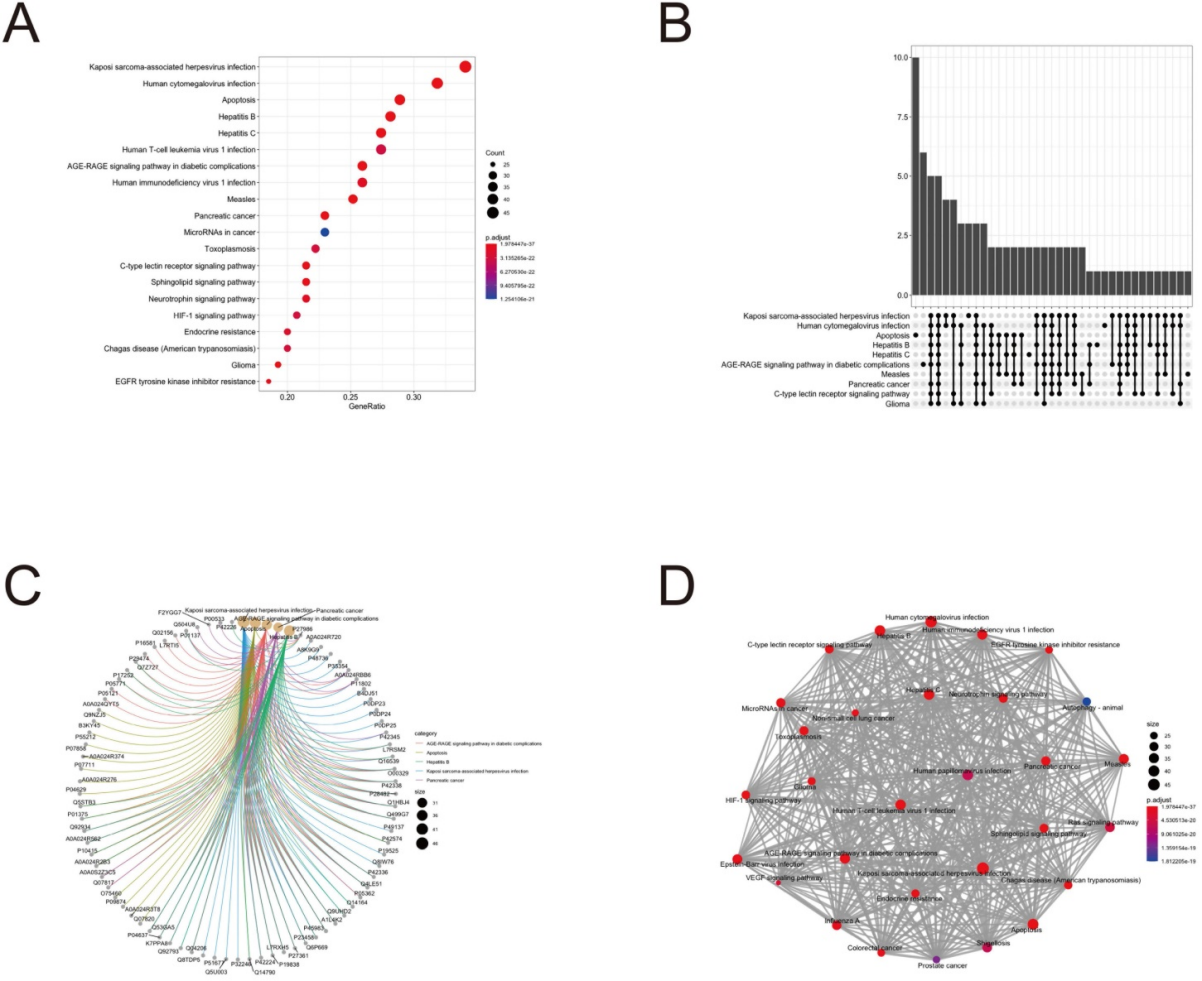
KEGG pathway analysis of potential therapeutic target genes.

To further clarify the functions of these 88 target genes, the results of GO and KEGG enrichment analysis were performed and presented by the R package “clusterProfiler”. The top 3 most enriched GO of differential genes were response to lipopolysaccharide, response to molecule of bacterial origin and positive regulation of cytokine production; membrane raft, membrane microdomain and membrane region; protein serine/threonine kinase activity, phosphatase binding and protein phosphatase binding by GO-BP, GO-CC and GO-MF, respectively (Fig. 3 and 4). Meanwhile, as shown in Figure 5, differential genes were most enriched in Kaposi sarcoma-associated herpes virus infection, human cytomegalovirus infection and apoptosis by KEGG pathway analysis. Each kind of the graphs represent different emphasis of the GO enrichment and KEGG pathway analysis. The bubble graphs (Fig. 3A, C and E; Fig. 5A) show the most significant GO terms/KEGG pathways, and also show the size of gene sets; the map graphs (Fig. 4B, D and F; Fig. 5D) integrate the GO terms/KEGG pathways and overlapped genes as a net, and the overlapped gene sets tend to occur in clusters; the gene concept network graphs are the network graphs that show the relationships and complicated associations between genes and the GO terms/KEGG pathways. These graphs (Fig. 4A, C and E; Fig. 5C) place emphasis on the details of the genes and GO terms/KEGG pathways, while the others place emphasis on the overlapping condition between genes of different gene sets involved (Fig. 3B, D and F; Fig. 5B).

### GeneMANIA analysis

**Figure 6:**
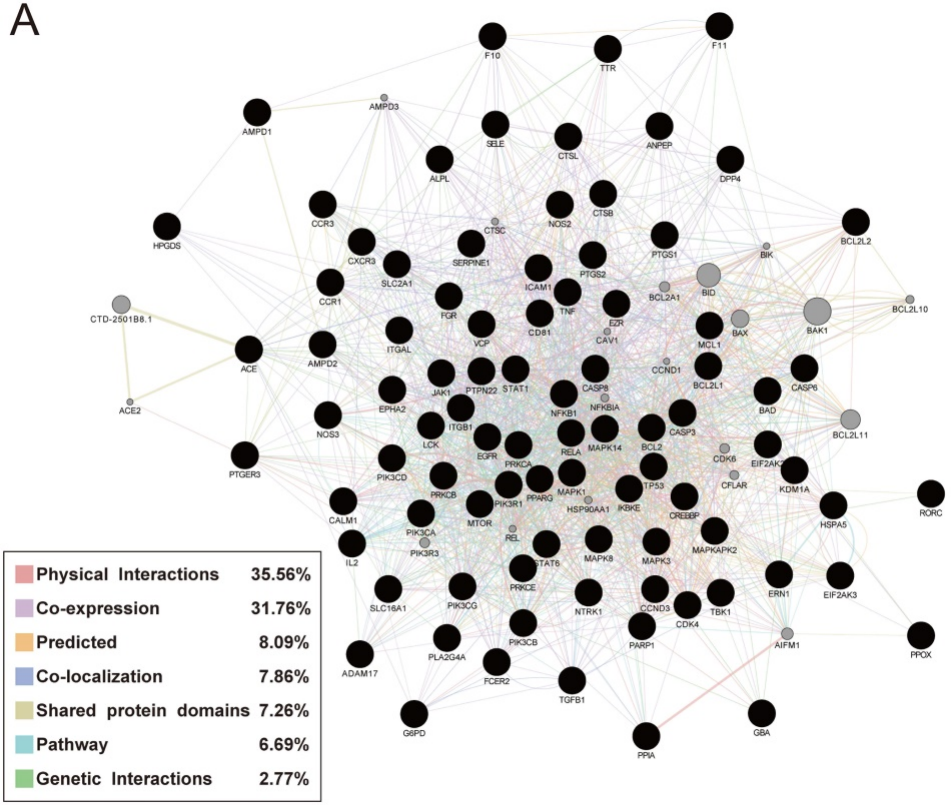
Protein network of common targets of Jingyin granule and COVID-19.

Performing GeneMANIA analysis to verify the relationship of the 88 targets and their interacting proteins, 35.56% of these genes had physical interactions, 37.76% displayed co-expression characteristics, 7.86% exerted co-localization, 7.26% shared the similar protein domains, 6.69% were involved in the biological pathway and 2.77% possessed genetic interactions (Fig. 6).

## Discussion

In the epidemic prevention and control of COVID-19, traditional Chinese medicine has shown its unique advantages, and Jingyin granule and other traditional Chinese medicine have been recommended in the treatment of COVID-19 by National Health Commission and National Administration of Traditional Chinese Medicine. In this study, we aim at identifying the main efficient ingredients of Jingyin granule and their potential therapeutic target genes in treating COVID-19.

Poor pharmacokinetics and toxicity, which could lead to side effects in the treatment of diseases and limitation of clinical usage, are the most important reason for the drug discovery and development delay or stagnation. In this way, more and more researchers believe that inherent property of the drug in the discovery process should be prioritized 23. Computer simulation can provide predictions of targets and pharmacokinetics, as well as pharmaceutical metabolism and intrinsic toxicity 24.

Among the rules of drug discovery nowadays, Lipinski's rule is a widely used rule to identify drug properties aiming to discover more convenient oral delivery and higher bioavailability drugs 25. This rule now becomes fundamental for drug discovery and selection.

In drug discovery, identification of drug target gene is the first and foremost step. Recently a variety of active compounds or drugs extracted from Chinese herbs are verified to interact with a variety of genes or related proteins 26. Numerous computational target identification methods have been discovered, among which databases for target prediction of small molecule are commonly used. In this study, 88 targets of Jingyin granule were identified using SwissTargePrediction database. The approach of GeneMANIA database gives overview and details of the gene and protein interactions. Interestingly, in this study we demonstrated that Jingyin granule could directly target the ACE gene, and ACE protein shared similar domain with ACE2, which had been identified as one of the most important targets that assist SARS-CoV-2 in accessing cells.

We identified the role as inflammatory regulator for Jingyin granule. For thousands of years, Flos lonicera, one of the widely used herbs in traditional Chinese medicine, has been shown to have the anti-inflammatory effect in treating upper respiratory tract infections. Luteolin, a compound extracted from Flos lonicera, has been proved to show a lot of biological activities, such as antioxidative and anti-inflammatory activities 27. Rui Yang *et al* also demonstrated that Licorice and its natural compounds have demonstrated anti-inflammatory activities 28. Accordance with these results, the results of GO and KEGG analysis revealed that Jingyin granule could also regulate immunoreaction.

The network of ingredients-targets also revealed that Jingyin granule has a variety of potential targets and therefore it might possess various pharmaceutical activities. NOS2, ADAM17, CDK4, MAPK14 and MAPK1 were involved in the top 3 GO-BP enrichment analysis. Nitric oxide (NO) has been validated as a mediator and regulator of inflammatory responses and microbial products or proinflammatory cytokines which could induce the expression of NOS2 in a large number of inflammatory and tissue cells 29. Alexandros Nicolaou *et al* demonstrated that ADAM17 may cleave membrane-bound TNFα and TNFR2, thus played a protective role in preventing overactivation of endogenous TNFR2 signaling in cells of the vasculature 30. Ling Zhang *et al* found that Qingjie Fuzheng Granule attenuated the intestinal mucositis and diarrhea induced by 5-FU via elevating CDK4 expression to promoting cell proliferation 31. Hua She *et al* found that LPS, the notable inflammatory mediator, could be sensed by the stress kinase MAPK14/p38a in microglia, which then directly phosphorylates and inhibits ULK1, inhibit autophagy and lead to a regular and full immunoreaction 32. Johanna C Sierra et al found that in gastric epithelial cells, activation of mitogen-activated protein kinase 1/3 (MAPK1/3) and activator protein 1 could be blocked by gefitinib, thus leading to chemokine synthesis dysfunction and inflammation inhibition 33. Multiple therapeutic target medicine, such as mixture of ingredients or compounds, are more effective in the treatment of complicated diseases. Hence, Jingyin granule could be a superb resource for future drug discovery.

## Conclusion

In summary, Jingyin granule is an active compound of herbs or a superb mixture of ingredients that could be further studied to discover a multiple target medicine with anti-infection effects. By using bioinformatic methods, this study provides a novel insight into the investigation of Jingyin granule, one of the widely used traditional Chinese medicine and offers an overview of its application in future clinical application.
